# Substrate-Selective Adhesion of Metal Nanoparticles to Graphene Devices

**DOI:** 10.1021/acs.jpclett.3c01542

**Published:** 2023-07-11

**Authors:** Patrick J. Edwards, Sean Stuart, James T. Farmer, Ran Shi, Run Long, Oleg V. Prezhdo, Vitaly V. Kresin

**Affiliations:** † Department of Physics and Astronomy, University of Southern California, Los Angeles, California 90089-0484, United States; ‡ Physical Sciences Laboratories, The Aerospace Corporation, 355 S. Douglas St., El Segundo, California 90245, United States; § College of Chemistry, Key Laboratory of Theoretical and Computational Photochemistry of Ministry of Education, Beijing Normal University, Beijing 100875, China; ∥ Department of Chemistry, University of Southern California, Los Angeles, California 90089, United States

## Abstract

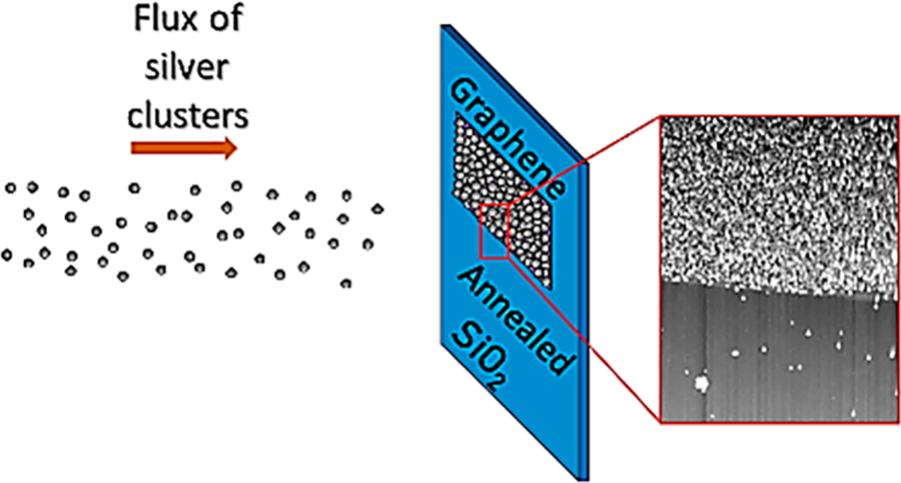

Nanostructured electronic devices, such as those based on graphene, are typically grown on top of the insulator SiO_2_. Their exposure to a flux of small size-selected silver nanoparticles has revealed remarkably selective adhesion: the graphene channel can be made fully metallized, while the insulating substrate remains coverage-free. This conspicuous contrast derives from the low binding energy between the metal nanoparticles and a contaminant-free passivated silica surface. In addition to providing physical insight into nanoparticle adhesion, this effect may be of value in applications involving deposition of metallic layers on device working surfaces: it eliminates the need for masking the insulating region and the associated extensive and potentially deleterious pre- and postprocessing.

Graphene has been extensively explored as a material in microelectronic devices, particularly in field effect transistor (FET) architecture. Its gapless semimetal nature enables fabrication of devices exhibiting ambipolar conduction with high carrier mobilities tunable via electrostatic gating. Additionally, the electronic structure of the devices’ exposed conducting graphene channel has been shown, for better or worse, to be easily affected by surface adsorbates. This fact has led to many studies aiming to use adsorbates to modify the carrier populations or bandgap of graphene FETs in order to adapt these devices to various applications in a controlled way (for examples, see refs (1−6)).

For the purpose of device modification, metallic nanoparticles and nanoclusters display many properties that make them suitable as graphene surface dopants. Their electron affinities and ionization energies are material- and size-dependent, making it possible to vary the amount of charge accepted from or donated to the graphene device.^7,8^ Nanoparticles possess strong optical resonances, enabling photoinduced charge transfer into the device^9,10^ and a range of other plasmonics-based graphene device applications^11−13^ such as photocatalysis, solar energy conversion, photodetection, and surface-enhanced Raman scattering. Furthermore, they can serve as anchors for external adsorbates, enhancing the devices’ functionality as sensors.^14−17^ Another interesting application is the capability of the graphene FET channel to detect the superconducting transition at the nanoscale.^18−20^

Bare metal nanoparticles deposited from the gas phase represent a particularly promising category of dopants. In contrast to their colloidally derived counterparts, gas phase particles do not require surface ligands used to prevent coalescence in solution. As such, there is no influence or contamination from ligands or their remnants and no need for harsh postprocessing required for their removal. Furthermore, there exist methods (such as the magnetron sputtering/condensation source utilized in the work described below) of producing directed beams of nanoparticles of a variety of materials, comprising either single elements or alloys with controllable species ratios.^21−23^ The nanoparticles in the beam are typically electrically charged, making it possible to filter them by size using mass spectrometry, measure their flux for accurate dosing, and adjust their kinetic energy for surface soft landing (or for energetic implantation if desired).

However, there is a drawback to decorating graphene channels by using a flux of nanoparticles, especially if multiple devices are fabricated on a single oxide wafer: upon exposure to the beam, the entire exposed surface, including the insulating region, may become metallized by a nanoparticle film. The conventional solution would employ lithographic techniques in which the devices are covered with a resist, a mask is developed in it so as to expose only the devices’ graphene channels, and the resist is removed after nanoparticle deposition. Unfortunately, lithographic resists used to pattern devices have been shown to alter the electronic properties of graphene and have proven to be very difficult, if not impossible, to fully remove once introduced. Furthermore, the available methods, such as acid treatments^24^ and high-temperature baking,^25^ risk damaging the dopant nanoparticles, substantially and uncontrollably altering their properties and their effect on the device.

In this work, we describe beam deposition of silver nanoparticles onto graphene on a silicon dioxide substrate. We discovered that when the devices are carefully cleaned prior to deposition, the particles almost exclusively coat the graphene and not the surrounding SiO_2_. As a consequence, graphene devices can be decorated and doped by metal nanoparticles and nanoclusters of variable sizes without the need of *any* postfabrication lithographic patterning.

Devices were fabricated from a commercially produced wafer of graphene grown by chemical vapor deposition (CVD) on a Si/SiO_2_ substrate with a 285 nm oxide layer (Grolltex). A rectangular channel was etched out of the graphene layer, uncovering the surrounding silica surface. Details of the procedure and an image of the device can be found in the Supporting Information. Prior to nanoparticle deposition, the devices were baked at 500 °C for 24–36 h in a 10^–10^–10^–9^ mbar vacuum chamber to remove residues of EBL resist (poly(methyl methacrylate) resin, PMMA) and other possible contaminants from the surface.^25^

For trials that involved only blank silica surfaces, Si/SiO_2_ wafers (MTI Corp.) were diced and cleaned via successive 30 min ultrasonications in acetone (twice), methanol (twice), isopropyl alcohol (twice), and deionized water. During sonication, these blocks (“dies”) were held in covered test tubes to prevent any particulates from collecting on the liquid meniscus and potentially transferring to the dies upon removal. As will be demonstrated, although acetone treatment is considered standard for PMMA removal,^26−28^ even this thorough procedure leaves some “sticky islands” on the surface which affect its adsorption properties.

A nanoparticle beam is produced using a DC magnetron gas aggregation source.^22,29^ This system consists of three segments: a gas-aggregation source chamber, a mass filter, and a deposition chamber. The base pressure in all chambers prior to deposition was approximately 10^–9^ mbar. In the source (Mantis Nanogen), a silver target (99.99%, ACI Alloys) is sputtered by an argon plasma at a power of 15–25 W. The resulting metal vapor condenses into nanoparticles while being transported inside a liquid nitrogen-cooled aggregation region by a flow of inert argon and helium gas. The data presented below were compared for various ratios of the flow rates of Ar/He through the source (140/70, 140/140, 70/70, 70/140, and 70/10, all values in sccm), and the conclusions were independent of this ratio. During operation, because of this gas flow the source and deposition pressures rose to ∼10^–4^ and ∼10^–5^ mbar, respectively. Because of the configuration of the magnetron block, the majority of nanoparticles are negatively charged.^30,31^

Upon exiting the condensation source, the resulting beam passes through a quadrupole mass filter (Mantis MesoQ) and enters the deposition chamber, where samples, mounted on a linear translation stage, can be exposed to it one at a time. The beam is collimated by an aperture, and the nanoparticle ion flux is determined with the help of a picoammeter (Keithley 6487). This establishes the exposure time required for the desired coverage.

After deposition, surface imaging was conducted via atomic force microscopy (AFM, Asylum Research Cypher ES). In order to avoid disturbing the weakly bound nanoparticles, the cantilever drive amplitude and set point were tuned to the regime of imaging using long-range attractive forces.^32^ Scanning electron microscopy (SEM, FEI Nova NanoSEM 450) verified the results as described below.

To gain insight into the expected coverage of nanoparticles on the graphene FETs, initial depositions were performed on separate small wafers of baked CVD graphene and of cleaned Si/SiO_2_ that were mounted on the same sample puck and exposed to the beam simultaneously. The mass spectrometer was set to select Ag nanoparticles of 6–8 nm diameter ((∼7–15) × 10^3^ atoms) for deposition, at nominal coverages ranging from 0.05 to 0.2 of a monolayer. The estimated 140 m/s velocity of the beam^33^ implies a deposition energy of ∼10 meV per atom. To rule out any possible role of beam profile inhomogeneity, some of the samples also were prepared with the wafers interchanged.

In every single trial, the observed density of nanoparticles on graphene significantly exceeded that on SiO_2_, even though they were exposed to the same beam flux. Figure 1 contains representative comparison images of the SiO_2_ (a) and graphene (b) surfaces.

**Figure 1:**
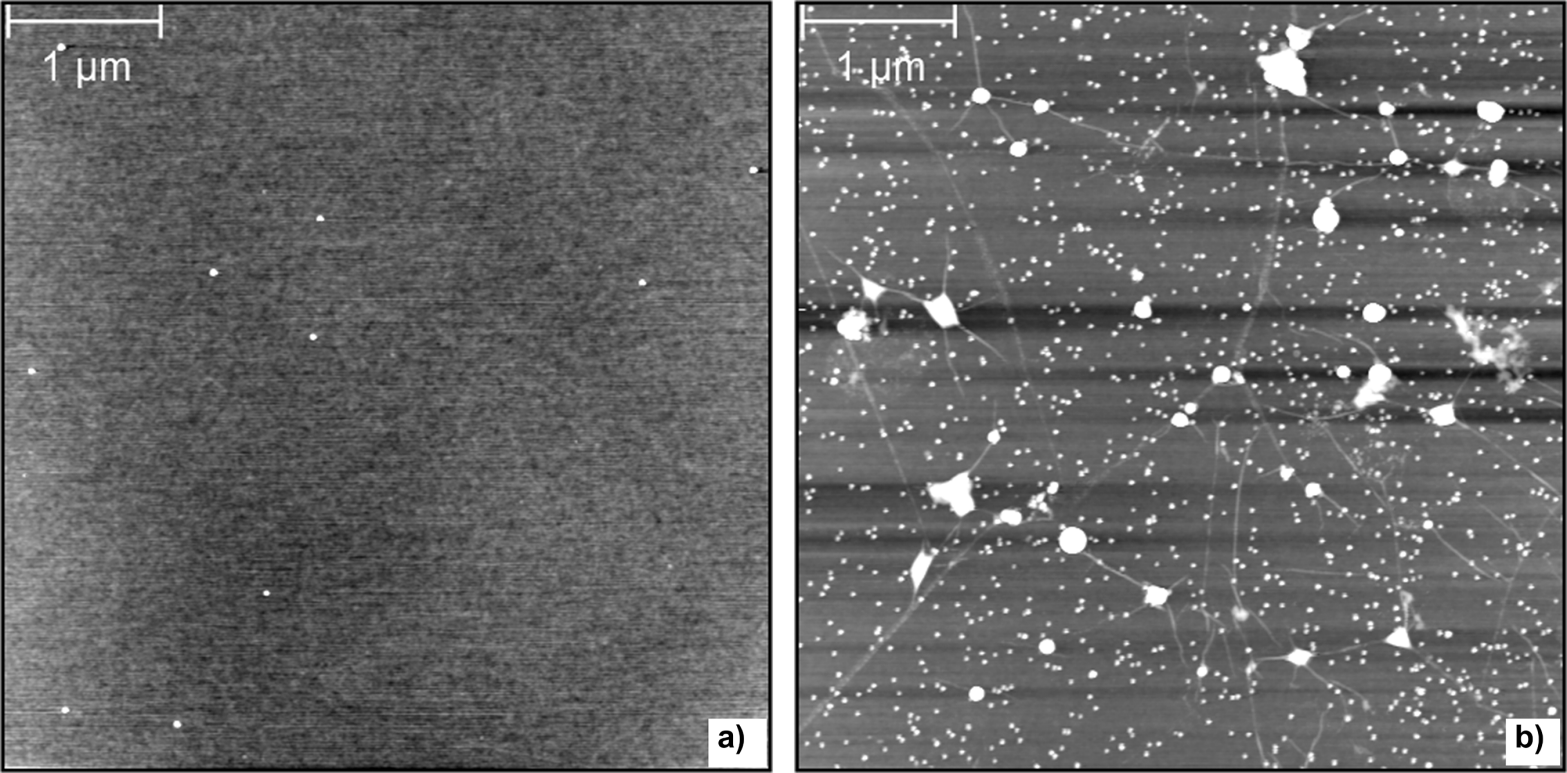
AFM imaging comparison of 8 nm Ag nanoparticle deposition on SiO_2_ (a) and graphene (b) under the same deposition conditions. The scan width of all images is the same, to illustrate the manyfold increase in nanoparticle density adsorbed on the graphene samples. (The larger white blotches on the graphene surface are PMMA patches which collected at crack defects, common to CVD graphene, and did not fully desorb during the baking process.)

To confirm the AFM scan settings and rule out any potential bias due to the tip–particle interaction, these observations were verified by SEM imaging. Figure 2a reveals that nanoparticles appear only in those areas of the SiO_2_ substrate where some surface contamination exists, plainly visible as a filmlike constrast around the dark nanoparticle dots. As mentioned above, these patches represent post-lithography or post-solvent residue which outlasted the cleaning process. On the other hand, clean SiO_2_ regions contain no adsorbed nanoparticles.

**Figure 2:**
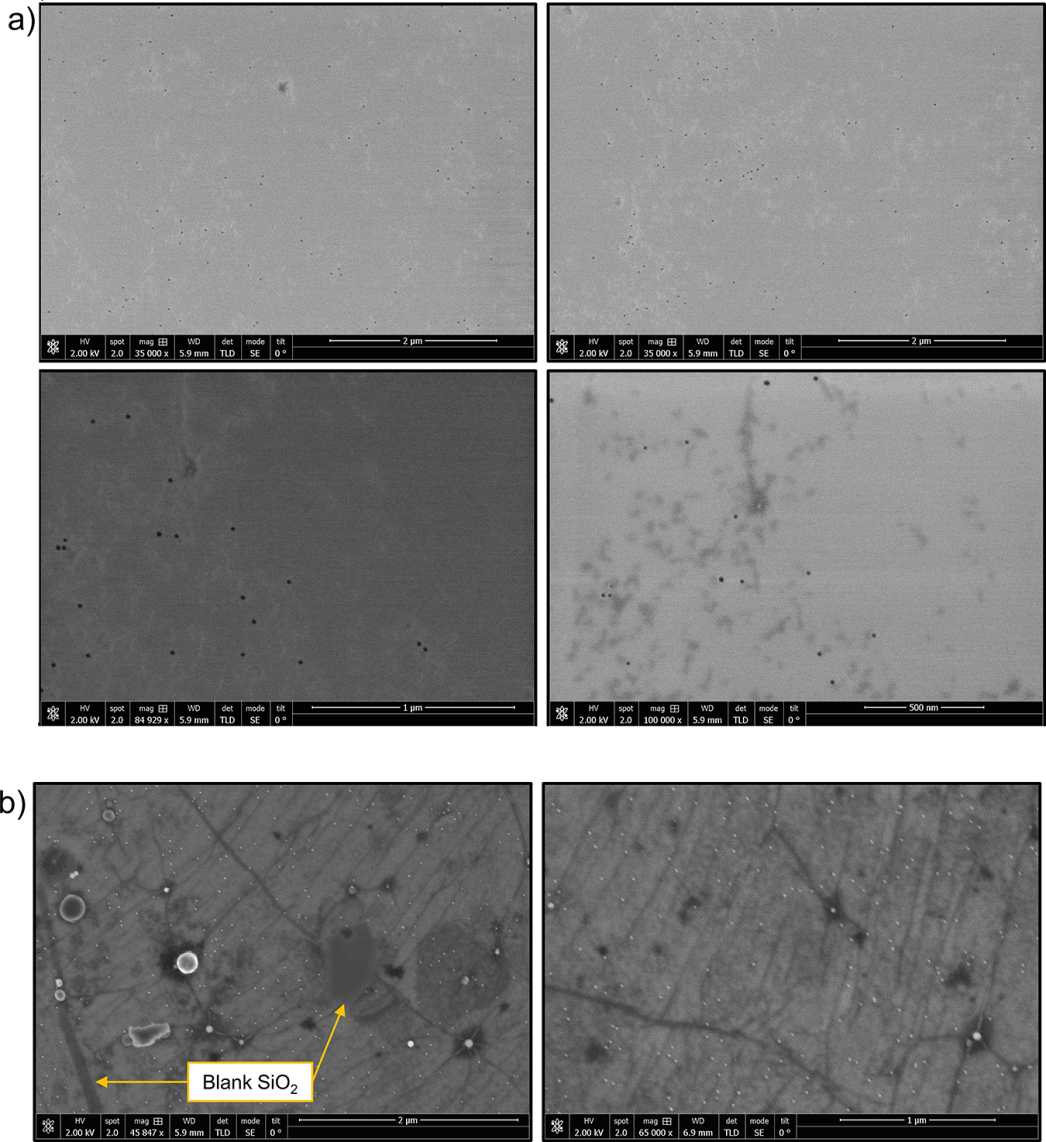
(a) SEM characterization of SiO_2_ surfaces after Ag nanoparticle deposition, confirming that the particle adsorption is low and documenting that it is confined to areas containing splotches of surface lithography residue. (b) SEM characterization of graphene surface after 0.1 monolayer Ag nanoparticle deposition. Ag clusters are observed on the graphene surface as small white dots, and a complete absence of deposition is noted along the exposed SiO_2_ underlayer.

The adjoining graphene samples were also examined (Figure 2b), confirming significantly greater nanoparticle coverage in every sample. Additionally, in this figure one finds occasional gaps in the graphene (crack defects or empty pits), which expose the underlying SiO_2_. These patches, which are residue-free, again show no sign of nanoparticle attachment.

Figure 3 shows the result of nominally 20% coverage of 10–20 nm silver nanoparticle onto a graphene FET device without a prebaking treatment. Consistent with the previous results, AFM imaging reveals a very large contrast between the nanoparticle populations on SiO_2_ and graphene areas of the sample, with the former population significantly lower than might have been anticipated and restricted to areas with residue contamination.

**Figure 3:**
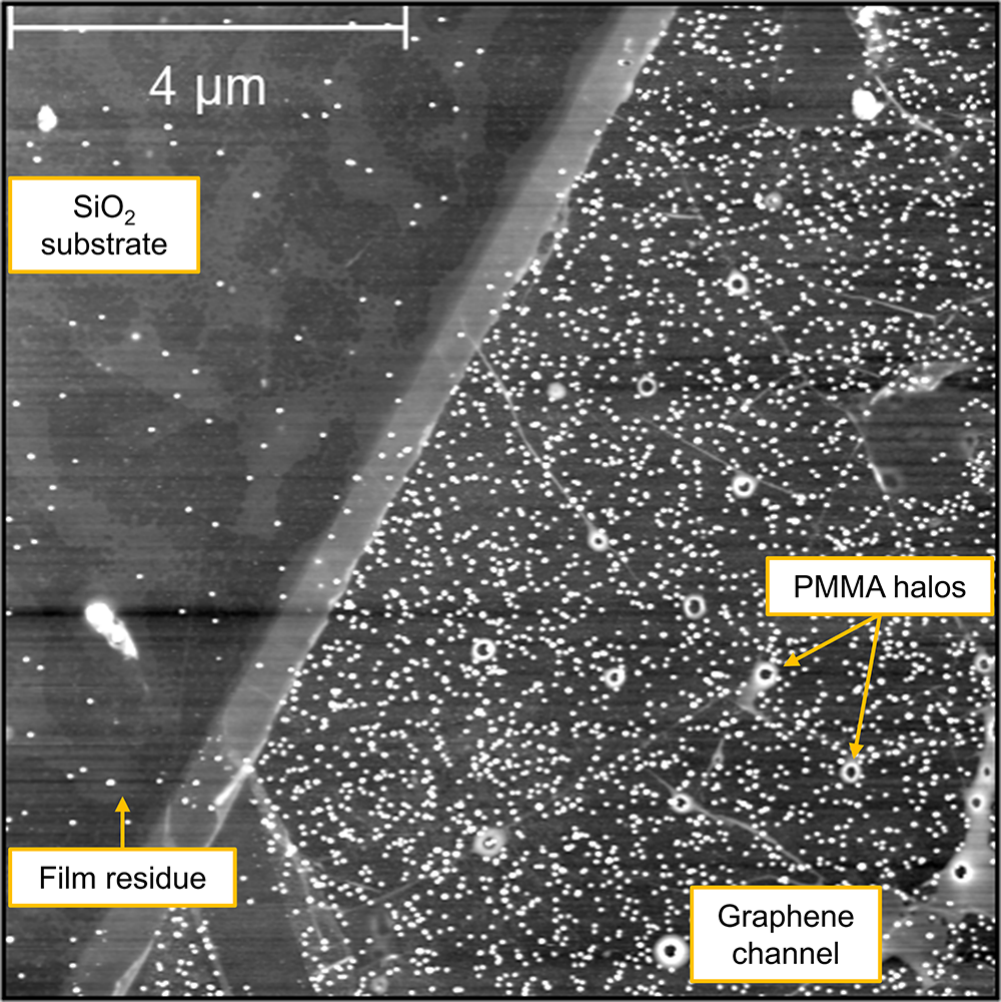
AFM imaging of dilute Ag nanoparticle deposition near the graphene channel boundary in the FET device. Again, a large population difference is observed between the two substrates. Without heat treatment, islands of PMMA residue persist on the surface, and once again, nanoparticles on SiO_2_ are observed only on them and not on pristine SiO_2_.

Finally, we explored whether the effect remains robust for heavier coatings, which would otherwise lead to complete metallization of the surface. We deposited 6–8 nm Ag nanoparticles onto graphene devices at nominal coverages of 3 and 6 monolayers. For comparison, in each deposition cycle one device was subjected to high vacuum bake-out prior to deposition, while another was left “as fabricated”. Figure 4a summarizes the results for 6 monolayer deposition, which is qualitatively the same as the 3 monolayer data shown in the Figure S2. The image clearly displays a stark contrast in surface coverage as a consequence of pretreatment. In the “as fabricated” device, the Ag deposition is continuous throughout, nearly to the point of obscuring the graphene step edge. In contrast, the precleaned device shows a heavy coverage of Ag on the graphene channel but few nanoparticles on the surrounding oxide. This figure underscores the main results of this work.

**Figure 4:**
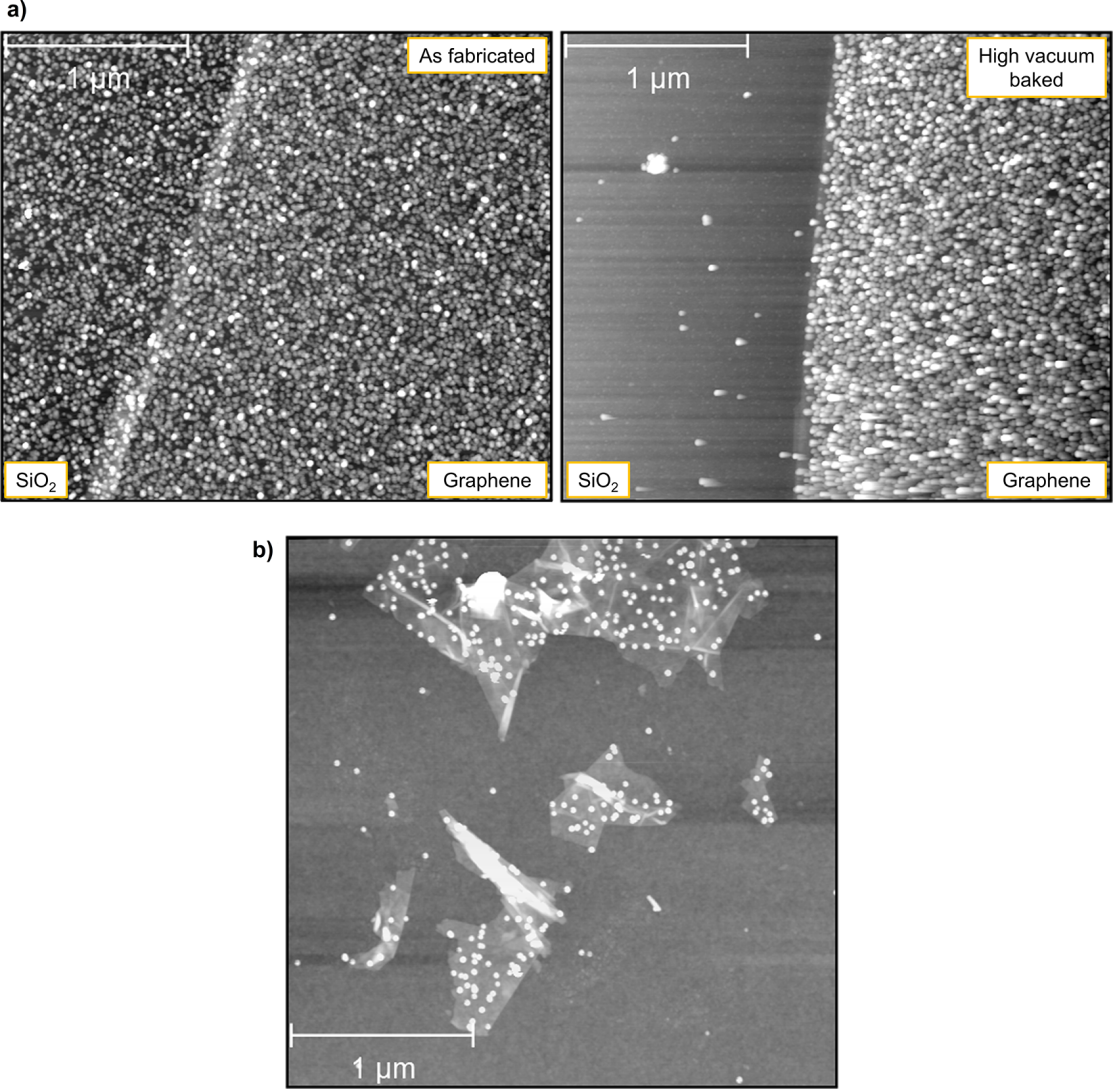
(a) AFM images of graphene FET devices after the deposition of 6 monolayers of Ag nanoparticles. An extremely high level of adhesion selectivity is observed for residue-free surfaces. (b) Nanoparticles are covering electrically isolated pieces of graphene but not the surrounding expanse of SiO_2_.

The images in Figures 2–4a uniformly point to the fact that incoming nanoparticles are disinclined to settle on contaminant-free SiO_2_ surfaces.

Because most of the nanoparticles arrive as ions, one may inquire whether the effect may be due to electrostatic charging of the oxide surface. The following observations verify that this is not the case. First, as was shown in Figure 2b, even patches of clean SiO_2_ surrounded by graphene remain free of nanoparticles despite being too small to set up a strong electric field by themselves. Second, we imaged an “inverse” configuration which had small and electrically isolated patches of graphene surrounded by SiO_2_ and found that the former became fully covered by nanoparticles, whereas the latter did not (see Figure 4b and the S3). If the oxide had been exerting a strong electrostatic repulsion, it would have prevented the nanoparticles from reaching the graphene. These images represent another striking illustration of the contrast in the nanoparticle adsorptivity between SiO_2_ and graphene. Third, we performed an analogous deposition experiment using mica, which also is an insulating surface, and found that, in contrast with silica, it becomes fully coated with nanoparticles (see Figure S4).

A purely diffusion-based mechanism for the lack of surface adsorption on a clean SiO_2_ surface also can be ruled out. Without any graphene to diffuse toward, one would expect to see some form of deposition on the oxide wafers. With graphene present, diffusion would lead a pile-up of nanoparticles along the edge boundaries,^31,34,35^ which is not observed in any images (see an additional illustration in Figure S3).

We can therefore infer that the behavior of silver nanoparticles on clean SiO_2_ surfaces derives from the very weak attraction between the two.

This conclusion was further supported by the observation that such nanoparticles were easily lifted off when the AFM was switched to contact-mode nanomanipulation. As described in the Supporting Information, in this mode we observed nanoparticles desorbing from pretreated silica surfaces in favor of adhering to an iridium-coated tip, something not seen upon deposition onto unbaked devices.

Taken as a whole, the results presented above point to the treatment of the SiO_2_ surface prior to deposition as the dominant factor in adsorption. They suggest that clean SiO_2_ is inherently uninterested in attaching silver nanoparticles.

Poor adhesion, though not in this extreme, has been observed in atomic deposition of silver thin films onto oxide surfaces.^36−38^ It is common practical knowledge in the device fabrication industry that when depositing Ag or Au electrical contact pads onto silicon oxide wafers, it is necessary first to deposit a thin intermediary “adhesion layer” such as Cr or Ti.^36^ Without it, noble atom films degrade in ambient conditions over time and eventually delaminate from the surface.^37^ It has been proposed to make use of this phenomenon for fabricating multilevel interconnects in semiconductor devices.^39^

While they are impermanent, atomic layers do coat silica surfaces at least temporarily. This is in contrast to the present nanoparticle deposition data; therefore, size effects also must play a role. This is consistent with the behavior of atomic noble-metal films: with time these films coarsen and form metal domains which then separate from the SiO_2_ surface.^37^ Thus, the phenomenon reported in our work involves an interplay between an inherently weak attractive force and finite-size effects. These factors have been explored by the model calculations described below.

In order to elucidate the atomistic origin of the observed effect within a practicable calculation, we compute the energy of interfacial binding between a silver nanoparticle and graphene and between the nanoparticle and SiO_2_ surfaces of different roughness. The binding energy
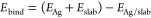
is calculated by subtracting the energy of the interacting system (*E*_Ag/slab_) from the sum of the energies of the bare slab (*E*_slab_), i.e., graphene or SiO_2_, and the nanoparticle. A positive binding energy reflects stronger chemisorption following the usual convention.^40,41^

To estimate the strength of the interfacial interaction, we select a pyramidal Ag_20_ nanocluster, which represents a typical model system,^42−45^ interfaced with either a 6 × 6 (001) graphene sheet or a 3 × 4 (001) SiO_2_ surface. Details of the simulation are presented in the Supporting Information. The systems used in the simulations are smaller than the experimental systems due to computational limitations. Long-range forces may become more relevant for the larger nanoparticles, but the general conclusions are expected to be applicable.

The undercoordinated Si and O atoms are passivated with −OH and −H, respectively, given that the (001) surface of the α-quartz SiO_2_ crystalline structure is hydrophilic^46,47^ and is exposed to water vapor during substrate mounting. (Even if the sample remained in the high-vacuum chamber, water molecules would be present in sufficient quantities to promptly saturate the surface bonds.) This aspect is important because passivation reduces the particle–substrate bonding energy by a factor of 5–10.

Using the optimized geometries shown in Figure 5, we obtain the binding energies of 3.71 eV for Ag_20_/graphene (Figure 5a) and 0.39 1.39, and 0.91 eV for the Ag_20_ nanoparticles interfaced with smooth (Figure 5b), slightly rough (Figure 5c), and strongly rough (Figure 5d) SiO_2_ surfaces. The interaction is much stronger in the Ag_20_/graphene system than at the Ag_20_/SiO_2_ interface, rationalizing the experimental observations that Ag nanoparticles attach strongly to the graphene. Interestingly, the binding energy is rather sensitive to the roughness of the SiO_2_ surface. The smooth SiO_2_ surface gives the weakest interaction, 1 order of magnitude weaker than graphene. The interaction is most favorable when the SiO_2_ surface is slightly rough. Excessive roughness weakens the interaction.

**Figure 5:**
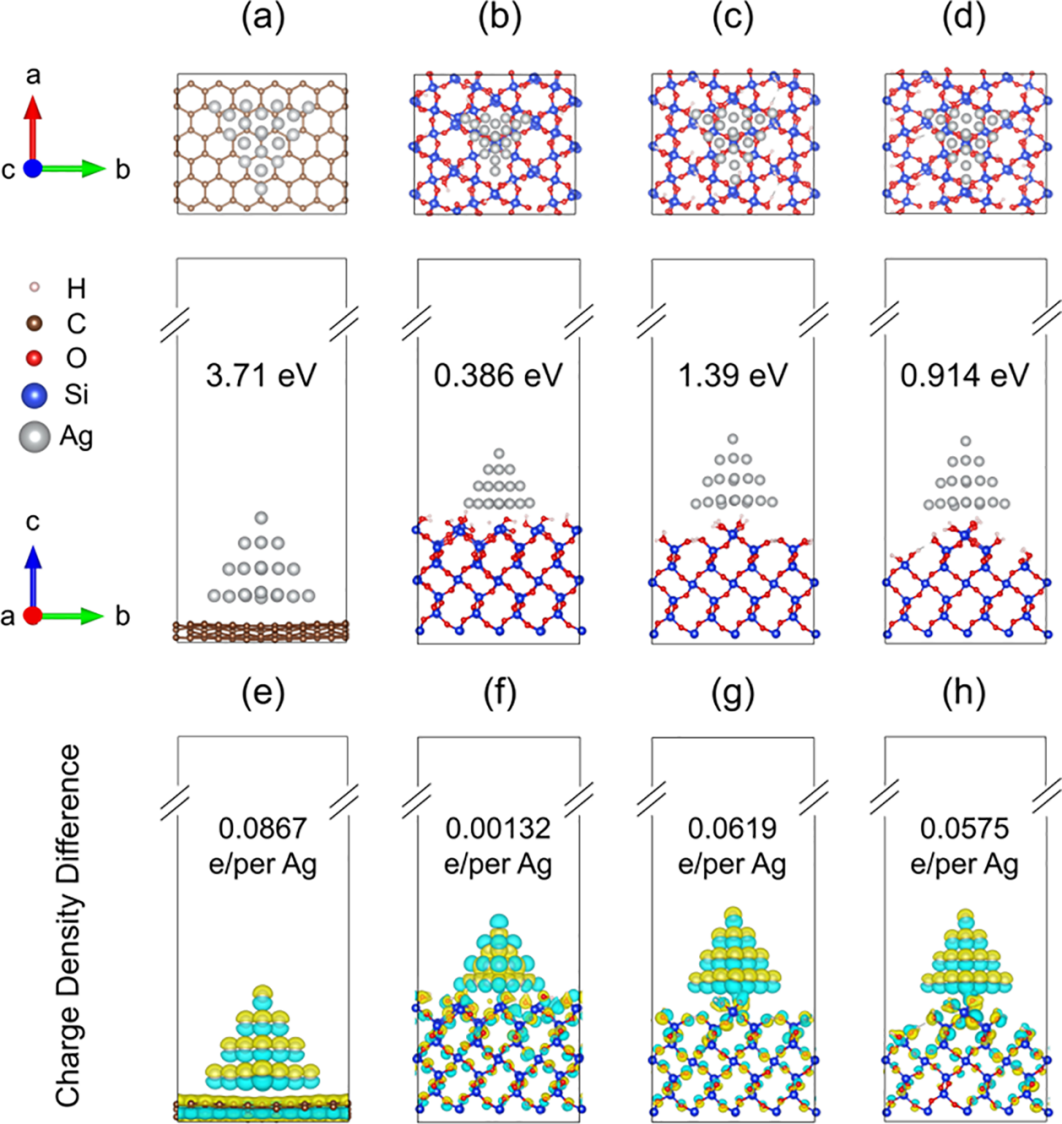
Top (top row) and side (middle row) views of the optimized geometries of (a) Ag_20_/graphene and Ag_20_ interfaced with (b) smooth, (c) slightly rough, and (d) strongly rough SiO_2_ surfaces. The binding energies are shown in the side-view panels. The charge density differences (isovalues: 0.06 au) for the four investigated systems are displayed in (e)–(h). Yellow: electron accumulation region. Blue: electron depletion region. The amount of charge transferred from Ag_20_ to the substrate is shown in the panels.

The strength of the interfacial interaction correlates well with the amount of charge transfer from Ag_20_ to the substrate, as characterized by the charge density difference and Bader charges.^48^ The electron accumulation and depletion regions are represented by yellow and blue colors, respectively (Figure 5e–h). This charge transfer from Ag_20_ to the substrate is also accompanied by notable charge redistribution within the cluster itself.

A significant amount of charge, 0.087*e* per Ag atom, is transferred from Ag_20_ to graphene (Figure 5e), which is associated with equilibration between the Fermi levels of the two systems. This leads to the strongest interfacial interaction. The amount of charge transfer between Ag_20_ and the SiO_2_ surface is larger for the rougher surfaces because of an increased local surface polarity that creates local electric fields. The smallest amount of charge is transferred from Ag_20_ to smooth SiO_2_, only 0.0013*e* (Figure 5f), correlating with the smallest binding energy. The electron loss per Ag atom is 0.062*e* and 0.058*e* for the slightly and strongly rough SiO_2_, also correlating with the binding energies. Thus the simulations demonstrate that variation in the SiO_2_ surface roughness can have a strong influence on the adsorption of silver nanoparticles.

In the present case, the post-treatment amorphous surface of SiO_2_ was measured to display a root mean square surface roughness of ∼0.2−0.3 nm. This places it near the category of a smooth surface. Furthermore, the nanoparticles used in this work are 6−10 nm in size, implying that adhesion forces are less influenced by charge transfer than the in the above model. Consequently, the experimentally observed contrast between graphene and silica surface coverage is concordant with the large difference between nanoparticle binding energies to these substrates, since weak binding enables the impinging nanoparticles to rebound upon surface impact.^49,51^

In conclusion, extremely high substrate selectivity was found for the coating of graphene devices by metal nanoparticles. Size-selective silver nanoparticle beam deposition onto properly precleaned devices decouples the coverage of the SiO_2_ and graphene surfaces without the need for additional processing steps. This selectivity offers a significant advantage because it avoids the contamination and delamination risk posed by lithography processes, which is significant for graphene. It also avoids the surface modifications of noble-metal nanoparticles required for the deposition from colloidal suspensions. This observation may be of value for the fabrication both of single devices (cf. bismuth nanoparticle-assembled interconnects on patterned photoresist templates^52,53^) and of wafers containing many devices in close proximity. Graphene structures covered by noble metal thin films have attracted interest,^54−57^ as have hybrid graphene–nanoparticle devices.

The data also highlight the high sensitivity of nanoparticle adsorption to surface contaminant removal. Model calculations of the interaction between silver nanoclusters and graphene or silica substrates confirm that particle binding to passivated SiO_2_ surfaces is significantly weaker than that to graphene. The calculations also call attention to the role played by charge transfer from the nanoparticle to the substrate and by the roughness of the latter. In future work, it will be valuablud e to further explore the role of size effects in the interplay between particle charging energies, surface roughness, contact areas, and electron transfer phenomena. It also will be of interest to extend the work to additional substrate materials and nanoparticle sizes, shapes, and compositions, as well as velocities, where nonmonotonic behavior of adhesion probabilities have been predicted.^50^

## Supporting Information Available

Device fabrication details, images of three-monolayer coverage, deposition on graphene patches, and deposition on mica, description of contact-mode AFM manipulation of nanoparticles, and computational details (PDF)

## References

[ref1] Schedin F.; Geim A. K.; Morozov S. V.; Hill E. W.; Blake P.; Katsnelson M. I.; Novoselov K. S. Detection of Individual Gas Molecules Adsorbed on Graphene. Nat. Mater. 2007, 6, 652–655. 10.1038/nmat1967. 17660825

[ref2] Varghese S. S.; Lonkar S.; Singh K. K.; Swaminathan S.; Abdala A. Recent Advances in Graphene Based Gas Sensors. Sensor. Actuat. B - Chem. 2015, 218, 160–183. 10.1016/j.snb.2015.04.062.

[ref3] Han W.; Kawakami R. K.; Gmitra M.; Fabian J. Graphene Spintronics. Nat. Nanotechnol. 2014, 9, 794–807. 10.1038/nnano.2014.214. 25286274

[ref4] Deng X.; Wu Y.; Dai J.; Kang D.; Zhang D. Electronic Structure Tuning and Band Gap Opening of Graphene by Hole/Electron Codoping. Phys. Lett. A 2011, 375, 3890–3894. 10.1016/j.physleta.2011.08.070.

[ref5] Amsterdam S. H.; Marks T. J.; Hersam M. C. Leveraging Molecular Properties to Tailor Mixed-Dimensional Heterostructures beyond Energy Level Alignment. J. Phys. Chem. Lett. 2021, 12, 4543–4557. 10.1021/acs.jpclett.1c00799. 33970639

[ref6] Abbas G.; Sonia F. J.; Jindra M.; Červenka J.; Kalbáč M.; Frank O.; Velický M. Electrostatic Gating of Monolayer Graphene by Concentrated Aqueous Electrolytes. J. Phys. Chem. Lett. 2023, 14, 4281–4288. 10.1021/acs.jpclett.3c00814. 37126786 PMC10184166

[ref7] Scheerder J. E.; Picot T.; Reckinger N.; Sneyder T.; Zharinov V. S.; Colomer J.-F.; Janssens E.; Van de Vondel J. Decorating Graphene with Size-Selected Few-Atom Clusters: A Novel Approach to Investigate Graphene–Adparticle Interactions. Nanoscale 2017, 9, 10494–10501. 10.1039/C7NR02217D. 28703819

[ref8] Akbari-Sharbaf A.; Ezugwu S.; Ahmed M. S.; Cottam M. G.; Fanchini G. Doping Graphene Thin Films with Metallic Nanoparticles: Experiment and Theory. Carbon 2015, 95, 199–207. 10.1016/j.carbon.2015.08.021.

[ref9] Nguyen K. T.; Li D.; Borah P.; Ma X.; Liu Z.; Zhu L.; Grüner G.; Xiong Q.; Zhao Y. Photoinduced Charge Transfer within Polyaniline-Encapsulated Quantum Dots Decorated on Graphene. ACS Appl. Mater. Interfaces 2013, 5, 8105–8110. 10.1021/am402182z. 23855339

[ref10] Dutta R.; Pradhan A.; Mondal P.; Kakkar S.; Sai T. P.; Ghosh A.; Basu J. K. Enhancing Carrier Diffusion Length and Quantum Efficiency through Photoinduced Charge Transfer in Layered Graphene–Semiconducting Quantum Dot Devices. ACS Appl. Mater. Interfaces 2021, 13, 24295–24303. 10.1021/acsami.1c04254. 33998798

[ref11] Li X.; Zhu J.; Wei B. Hybrid Nanostructures of Metal/Two-Dimensional Nanomaterials for Plasmon-Enhanced Applications. Chem. Soc. Rev. 2016, 45, 3145–3187. 10.1039/C6CS00195E. 27048993

[ref12] Grigorenko A. N.; Polini M.; Novoselov K. Graphene Plasmonics. Nat. Photonics 2012, 6, 749–758. 10.1038/nphoton.2012.262.

[ref13] Cherqui C.; Li G.; Busche J. A.; Quillin S. C.; Camden J. P.; Masiello D. J. Multipolar Nanocube Plasmon Mode-Mixing in Finite Substrates. J. Phys. Chem. Lett. 2018, 9, 504–512. 10.1021/acs.jpclett.7b03271. 29314843

[ref14] Wang T.; Huang D.; Yang Z.; Xu S.; He G.; Li X.; Hu N.; Yin G.; He D.; Zhang L. A Review on Graphene-Based Gas/Vapor Sensors with Unique Properties and Potential Applications. Nano-Micro Lett. 2016, 8, 95–119. 10.1007/s40820-015-0073-1. PMC6223682 30460270

[ref15] Ruffino F.; Giannazzo F. A Review on Metal Nanoparticles Nucleation and Growth on/in Graphene. Crystals 2017, 7, 219 10.3390/cryst7070219.

[ref16] Shtepliuk I.; Yakimova R. Computational Appraisal of Silver Nanocluster Evolution on Epitaxial Graphene: Implications for CO Sensing. ACS Omega 2021, 6, 24739–24751. 10.1021/acsomega.1c03577. 34604656 PMC8482456

[ref17] Cui S.; Mao S.; Lu G.; Chen J. Graphene Coupled with Nanocrystals: Opportunities and Challenges for Energy and Sensing Applications. J. Phys. Chem. Lett. 2013, 4, 2441–2454. 10.1021/jz400976a.

[ref18] Kessler B.; Girit Ç.; Zettl A.; Bouchiat V. Tunable Superconducting Phase Transition in Metal-Decorated Graphene Sheets. Phys. Rev. Lett. 2010, 104, 047001 10.1103/PhysRevLett.104.047001. 20366731

[ref19] Allain A.; Han Z.; Bouchiat V. Electrical Control of the Superconducting-to-Insulating Transition in Graphene–Metal Hybrids. Nat. Mater. 2012, 11, 590–594. 10.1038/nmat3335. 22609559

[ref20] Han Z.; Allain A.; Arjmandi-Tash H.; Tikhonov K.; Feigel’Man M.; Sacépé B.; Bouchiat V. Collapse of Superconductivity in a Hybrid Tin–Graphene Josephson Junction Array. Nat. Phys. 2014, 10, 380–386. 10.1038/nphys2929.

[ref21] Milani P.; Iannotta S. Cluster Beam Synthesis of Nanostructured Materials; Springer: Berlin, 1999.

[ref22] Gas-Phase Synthesis of Nanoparticles; Huttel Y., Ed.; Wiley-VCH: Weinheim, 2017.

[ref23] Cluster Beam Deposition of Functional Nanomaterials and Devices; Milani P., Sowwan M., Eds.; Elsevier: Amsterdam, 2020.

[ref24] Her M.; Beams R.; Novotny L. Graphene Transfer with Reduced Residue. Phys. Lett. A 2013, 377, 1455–1458. 10.1016/j.physleta.2013.04.015.

[ref25] Xie W.; Weng L.-T.; Ng K. M.; Chan C. K.; Chan C.-M. Clean Graphene Surface through High Temperature Annealing. Carbon 2015, 94, 740–748. 10.1016/j.carbon.2015.07.046.

[ref26] Yu Q.; Lian J.; Siriponglert S.; Li H.; Chen Y. P.; Pei S.-S. Graphene Segregated on Ni Surfaces and Transferred to Insulators. Appl. Phys. Lett. 2008, 93, 113103 10.1063/1.2982585.

[ref27] Reina A.; Jia X.; Ho J.; Nezich D.; Son H.; Bulovic V.; Dresselhaus M. S.; Kong J. Large Area, Few-Layer Graphene Films on Arbitrary Substrates by Chemical Vapor Deposition. Nano Lett. 2009, 9, 30–35. 10.1021/nl801827v. 19046078

[ref28] Li X.; Cai W.; An J.; Kim S.; Nah J.; Yang D.; Piner R.; Velamakanni A.; Jung I.; Tutuc E.; et al. Large-Area Synthesis of High-Quality and Uniform Graphene Films on Copper Foils. Science 2009, 324, 1312–1314. 10.1126/science.1171245. 19423775

[ref29] Haberland H.; Mall M.; Moseler M.; Qiang Y.; Reiners T.; Thurner Y. Filling of Micron-sized Contact Holes with Copper by Energetic Cluster Impact. J. Vac. Sci. Technol. A 1994, 12, 2925–2930. 10.1116/1.578967.

[ref30] Khojasteh M. Fabrication, Deposition, and Characterization of Size-Selected Metal Nanoclusters With a Magnetron Sputtering Gas Aggregation Source; University of Southern California: 2019.

[ref31] Johnson G. E.; Colby R.; Laskin J. Soft Landing of Bare Nanoparticles with Controlled Size, Composition, and Morphology. Nanoscale 2015, 7, 3491–3503. 10.1039/C4NR06758D. 25626391

[ref32] San Paulo A.; García R. High-Resolution Imaging of Antibodies by Tapping-Mode Atomic Force Microscopy: Attractive and Repulsive Tip-Sample Interaction Regimes. Biophys. J. 2000, 78, 1599–1605. 10.1016/S0006-3495(00)76712-9. 10692344 PMC1300757

[ref33] Khojasteh M.; Kresin V. V. Influence of Source Parameters on the Growth of Metal Nanoparticles by Sputter-Gas-Aggregation. Appl. Nanosci. 2017, 7, 875–883. 10.1007/s13204-017-0627-2.

[ref34] Francis G.; Kuipers L.; Cleaver J.; Palmer R. Diffusion Controlled Growth of Metallic Nanoclusters at Selected Surface Sites. J. Appl. Phys. 1996, 79, 2942–2947. 10.1063/1.361290.

[ref35] Carroll S.; Seeger K.; Palmer R. Trapping of Size-Selected Ag Clusters at Surface Steps. Appl. Phys. Lett. 1998, 72, 305–307. 10.1063/1.120719.

[ref36] Nicolet M.-A. Diffusion Barriers in Thin Films. Thin Solid Films 1978, 52, 415–443. 10.1016/0040-6090(78)90184-0.

[ref37] Khanna V. Adhesion–Delamination Phenomena at the Surfaces and Interfaces in Microelectronics and MEMS Structures and Packaged Devices. J. Phys. D Appl. Phys. 2011, 44, 034004 10.1088/0022-3727/44/3/034004.

[ref38] Romanyuk A.; Steiner R.; Mack I.; Oelhafen P.; Mathys D. Growth of Thin Silver Films on Silicon Oxide Pretreated by Low Temperature Argon Plasma. Surf. Sci. 2007, 601, 1026–1030. 10.1016/j.susc.2006.11.044.

[ref39] Aono T.; Iwasaki T.; Yoshimura Y.; Nakayama Y. Method for Fabricating Multilevel Interconnection Structures Using Differential Delamination Energies between Metal and Silicon Oxide Thin Films. Electr. Commun. Jpn. 2016, 99, 41–49. 10.1002/ecj.11823.

[ref40] Nakada K.; Ishii A. Migration of Adatom Adsorption on Graphene Using DFT Calculation. Solid State Commun. 2011, 151, 13–16. 10.1016/j.ssc.2010.10.036.

[ref41] Sorescu D. C.; Thompson D. L.; Hurley M. M.; Chabalowski C. F. First-Principles Calculations of the Adsorption, Diffusion, and Dissociation of a CO Molecule on the Fe (100) Surface. Phys. Rev. B 2002, 66, 035416 10.1103/PhysRevB.66.035416.

[ref42] Chu W.; Saidi W. A.; Prezhdo O. V. Long-Lived Hot Electron in a Metallic Particle for Plasmonics and Catalysis: *Ab Initio* Nonadiabatic Molecular Dynamics with Machine Learning. ACS Nano 2020, 14, 10608–10615. 10.1021/acsnano.0c04736. 32806073

[ref43] Rapacioli M.; Spiegelman F.; Tarrat N. Evidencing the Relationship between Isomer Spectra and Melting: The 20-and 55-Atom Silver and Gold Cluster Cases. Phys. Chem. Chem. Phys. 2019, 21, 24857–24866. 10.1039/C9CP03897C. 31539012

[ref44] Ren X.; Lin W.; Fang Y.; Ma F.; Wang J. Raman Optical Activity (ROA) and Surface-Enhanced ROA (SE-ROA) of (+)-(R)-Methyloxirane Adsorbed on a Ag_20_ Cluster. RSC Adv. 2017, 7, 34376–34381. 10.1039/C7RA04949H.

[ref45] Liu L.; Chen D.; Ma H.; Liang W. Spectral Characteristics of Chemical Enhancement on SERS of Benzene-like Derivatives: Franck–Condon and Herzberg–Teller Contributions. J. Phys. Chem. C 2015, 119, 27609–27619. 10.1021/acs.jpcc.5b05910.

[ref46] Gao W.; Xiao P.; Henkelman G.; Liechti K. M.; Huang R. Interfacial Adhesion between Graphene and Silicon Dioxide by Density Functional Theory with van Der Waals Corrections. J. Phys. D Appl. Phys. 2014, 47, 255301 10.1088/0022-3727/47/25/255301.

[ref47] Fan X.; Zheng W.; Chihaia V.; Shen Z.; Kuo J.-L. Interaction Between Graphene and the Surface of SiO_2_. J. Phys. - Condens. Mater. 2012, 24, 305004 10.1088/0953-8984/24/30/305004. 22713875

[ref48] Henkelman G.; Arnaldsson A.; Jónsson H. A Fast and Robust Algorithm for Bader Decomposition of Charge Density. Comput. Mater. Sci. 2006, 36, 354–360. 10.1016/j.commatsci.2005.04.010.

[ref49] Awasthi A.; Hendy S.; Zoontjens P.; Brown S. Reentrant Adhesion Behavior in Nanocluster Deposition. Phys. Rev. Lett. 2006, 97, 186103 10.1103/PhysRevLett.97.186103. 17155557

[ref50] Awasthi A.; Hendy S.; Zoontjens P.; Brown S.; Natali F. Molecular Dynamics Simulations of Reflection and Adhesion Behavior in Lennard-Jones Cluster Deposition. Phys. Rev. B 2007, 76, 115437 10.1103/PhysRevB.76.115437.

[ref51] Weir G. A Simple Conceptual Model for the Behaviour of an Impacting Rigid-Plastic, Spherical, Nano-Scale Particle. Curr. Appl. Phys. 2008, 8, 355–358. 10.1016/j.cap.2007.10.055.

[ref52] Reichel R.; Partridge J. G.; Natali F.; Matthewson T.; Brown S. A.; Lassesson A.; Mackenzie D. M. A.; Ayesh A. I.; Tee K. C.; Awasthi A.; Hendy S. C. From the Adhesion of Atomic Clusters to the Fabrication of Nanodevices. Appl. Phys. Lett. 2006, 89, 213105 10.1063/1.2387894.

[ref53] Partridge J.; Matthewson T.; Brown S. Bi Cluster-Assembled Interconnects Produced Using SU8 Templates. Nanotechnology 2007, 18, 155607 10.1088/0957-4484/18/15/155607.

[ref54] Rast L.; Sullivan T.; Tewary V. K. Stratified Graphene/Noble Metal Systems for Low-Loss Plasmonics Applications. Phys. Rev. B 2013, 87, 045428 10.1103/PhysRevB.87.045428.

[ref55] Yakubovsky D. I.; Stebunov Y. V.; Kirtaev R. V.; Voronin K. V.; Voronov A. A.; Arsenin A. V.; Volkov V. S. Graphene-Supported Thin Metal Films for Nanophotonics and Optoelectronics. Nanomaterials 2018, 8, 1058 10.3390/nano8121058. 30558333 PMC6316737

[ref56] Shtepliuk I.; Ivanov I. G.; Pliatsikas N.; Iakimov T.; Jamnig A.; Sarakinos K.; Yakimova R. Probing the Uniformity of Silver-Doped Epitaxial Graphene by Micro-Raman Mapping. Physica B 2020, 580, 411751 10.1016/j.physb.2019.411751.

[ref57] Chahal S.; Bandyopadhyay A.; Dash S. P.; Kumar P. Microwave Synthesized 2D Gold and Its 2D-2D Hybrids. J. Phys. Chem. Lett. 2022, 13, 6487–6495. 10.1021/acs.jpclett.2c01540. 35819242

