# Supplementary material for: Substrate-Selective Adhesion of Metal Nanoparticles to Graphene Devices
Source: J Phys Chem Lett. 2023 Jul 11;14(28):6414–21. doi: 10.1021/acs.jpclett.3c01542 (PMC10364134; doi:10.1021/acs.jpclett.3c01542)
Supplement: Supplementary file 1 — jz3c01542_si_001.pdf [file jz3c01542_si_001.pdf]

Supporting Information for

Substrate-Selective Adhesion of Metal Nanoparticles to Graphene Devices

Patrick J. Edwards[1,2], Sean C. Stuart[2], James T. Farmer[1], Ran Shi,[3] Run Long,[3] Oleg V. Prezhdo[1,4], Vitaly V. Kresin[1]

[1] Department of Physics and Astronomy, University of Southern California, Los Angeles, CA 90089-0484, USA

[2] Physical Sciences Laboratories, The Aerospace Corporation, 355 S. Douglas St., El Segundo, CA 90245, USA

[3] College of Chemistry, Key Laboratory of Theoretical and Computational Photochemistry of Ministry of Education, Beijing Normal University, Beijing 100875, China

[4] Department of Chemistry, University of Southern California, Los Angeles, CA 90089, USA

<u>Contents</u>

S-I Device fabrication

S-II Three-monolayer nanoparticle coverage

S-III Nanoparticle deposition on graphene patches

S-IV Nanoparticle deposition on mica

S-V Contact-mode nanomanipulation

S-VI Computational details

S-VII References

S-I. Device fabrication

Graphene FET devices were fabricated in a cleanroom environment at the Aerospace Corporation. Square 1 cm × 1 cm dies were cut from a wafer of CVD graphene grown on a Si/SiO_2 substrate. Each die was then patterned with a 5 × 5 array of four-probe graphene FETs via a multi-step lithography process. The rectangular graphene channel was defined by electron beam lithography (EBL) and etched out of the graphene layer by using an argon plasma. Then an additional EBL and electron beam physical vapor deposition (EBPVD) process was performed to deposit four Ti/Au (10nm/50 nm) contacts onto the channel surface, completing the device. An image of one FET is shown in Figure S1.

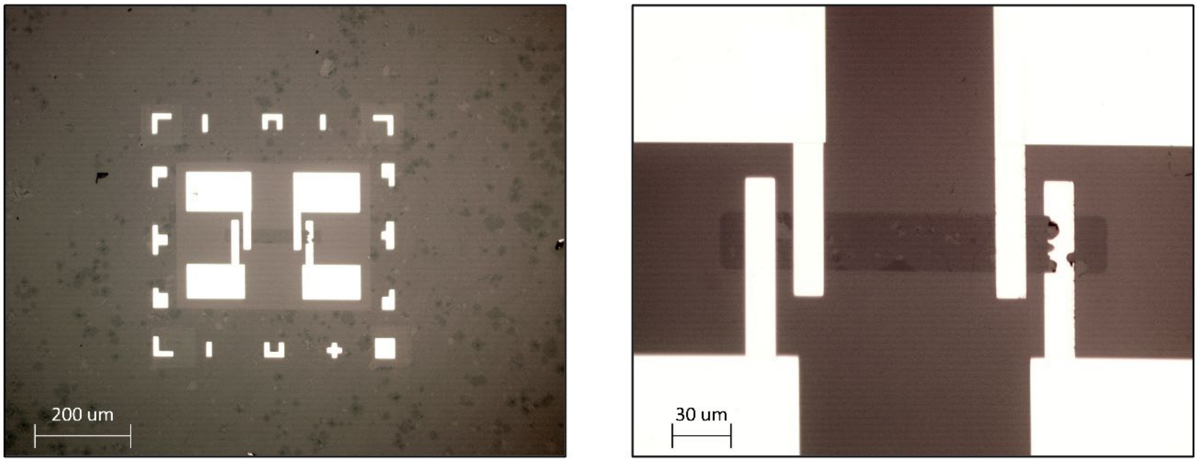

Figure S1. Optical microscopy imaging of a completed graphene device showing an isolated graphene channel (dark strip in the center of the image) on an SiO_2 substrate with Ti/Au surface contacts.

S-II. Three-monolayer nanoparticle coverage

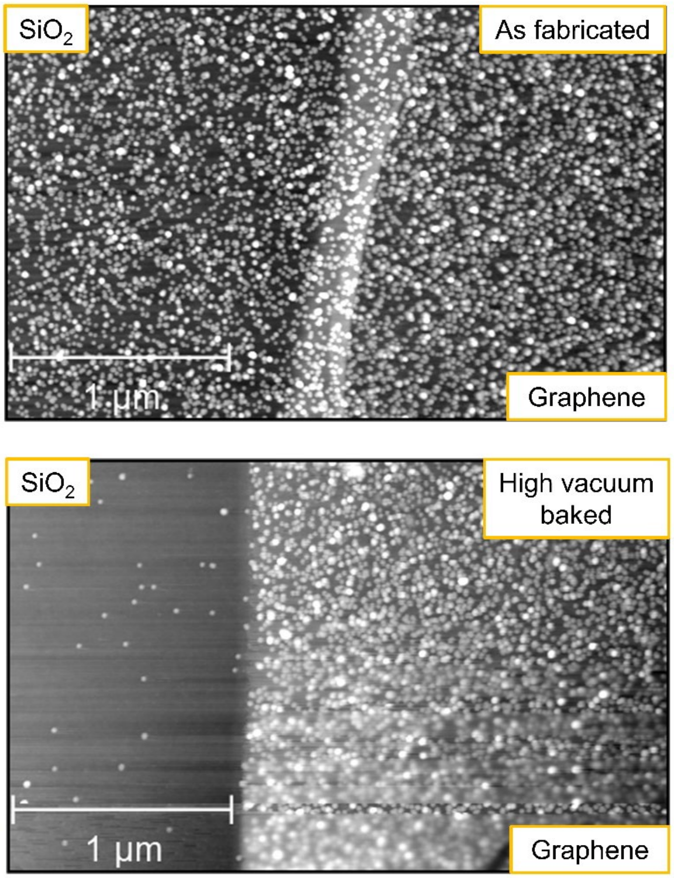

Figure S2. AFM images of graphene FET devices after the deposition of three monolayers of Ag nanoparticles. The effect of residue removal pre-treatment by device baking on the nanoparticle adhesion is clearly visible. (Loss of resolution at the bottom of the lower image is due to partial tip disengagement during scanning.)

S-III. Nanoparticle deposition on graphene patches

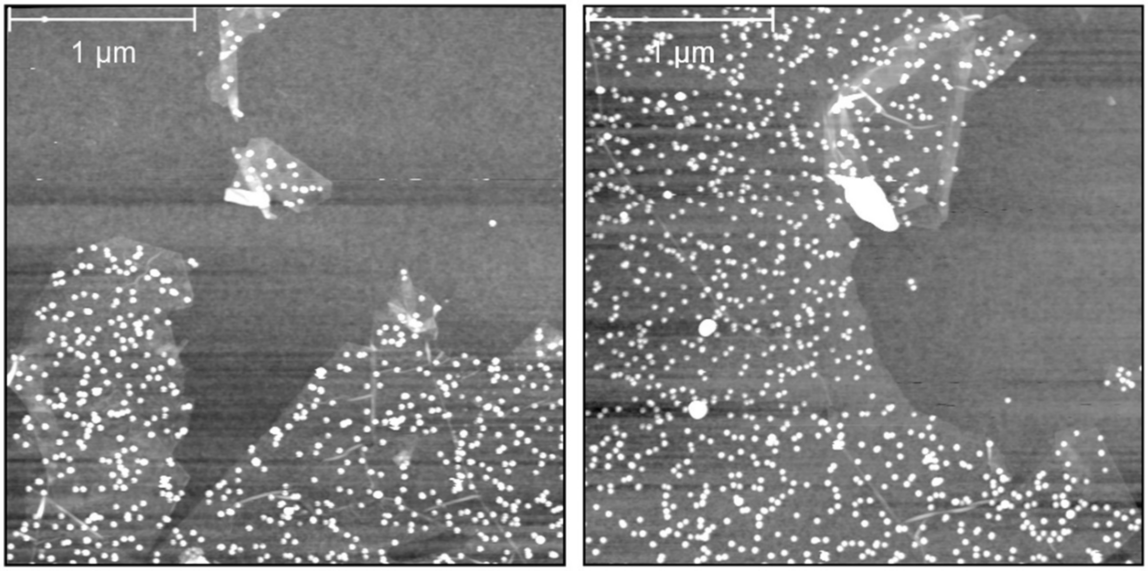

Figure S3. Silver nanoparticles cover electrically isolated islands of graphene but not the surrounding expanse of SiO_2. There also is no evidence of nanoparticle aggregation along the graphene step edges, and hence of any significant surface diffusion.

S-IV. Nanoparticle deposition on mica

A disc of mica (Ted Pella, optically flat grade V1) was cleaved in air and mounted adjacent to a wafer of CVD graphene in the same manner as the samples described in the main text. Analogously to the graphene substrate, the mica surface possesses an organized crystal structure and provides a very flat surface upon which to deposit nanoparticles.[S1, S2] However, analogously to silica, it is electrically insulating. Imaging shows that the mica and the graphene substrates become similarly covered.

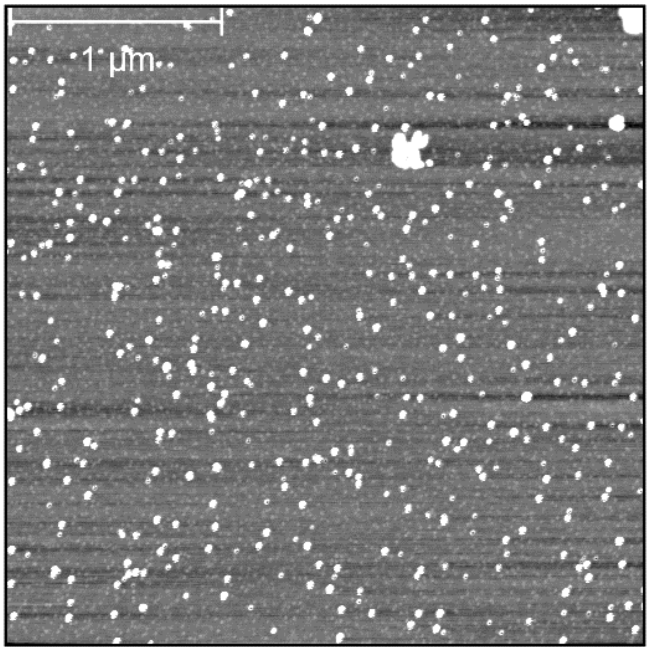

Figure **S4.** Insulating mica samples show a similar degree of nanoparticle coverage as the adjacent CVD graphene substrates.

S-V. Contact-mode nanomanipulation

Nanomanipulation experiments were conducted using Ir/Ti coated conductive silicon tips (Oxford Instruments ASYELEC.01-R2). The procedure is first to obtain a small (~1 μm^2) field-of-view image of the deposited nanoparticles near a graphene/SiO_2 step edge (see Figure S4 top left). This first image is acquired in the non-contact attractive regime, so as not to disturb the particles, as described in the main text. Once this image is obtained, the system allows for seamless transition to contact-mode imaging where custom tip deflection settings and paths can be preset by using the collected image as a reference (see the path overlay in Figure S4 top right). The paths are then traced out, in order, while the tip is in contact with the sample surface. For all contact traces the tip speed is set to the slowest possible scan rate of 5 nm/s, and the tip lifts off the surface when relocating from the end of one trace to the beginning of the next. After all contact traces are completed, the image area is finally rescanned in the non-contact mode to reveal the result of the manipulations. Figure S4 depicts a representative manipulation cycle and displays the range of observed outcomes.

Trace 1 shows the move of a nanoparticle off the graphene onto the cleaned SiO_2 surface.

Trace 2 demonstrates a frequent occurrence for a nanoparticle that had already been moved to the SiO_2 during a previous manipulation. This trace sought simply to move it along the oxide surface, but the post-manipulation image finds that the particle has been "erased" from the region (Figure S4 bottom). What happened is that the nanoparticle desorbed from the substrate and attached itself to the Ir/Ti coated tip. Such an outcome also has been observed while attempting to move nanoparticles from graphene to SiO_2 (akin to trace 1).

Finally, trace 3 relocated a cluster along the graphene toward the SiO_2 interface. A close inspection of the result reveals not one but two nanoparticles at the final location (highlighted by the blue box). Thus, it appears that the above tip-adsorbed nanoparticle has been released onto the graphene surface.

The attachment of nanoparticles to the tip was observed only when moving them along the clean SiO_2 substrate, and nanoparticle were never removed from the graphene. Additionally, nanoparticle redeposition was only observed onto the graphene surface. This suggests that while

the nanoparticle interaction with the tip is stronger than that with the oxide surface, the nanoparticle-graphene interaction is stronger still.

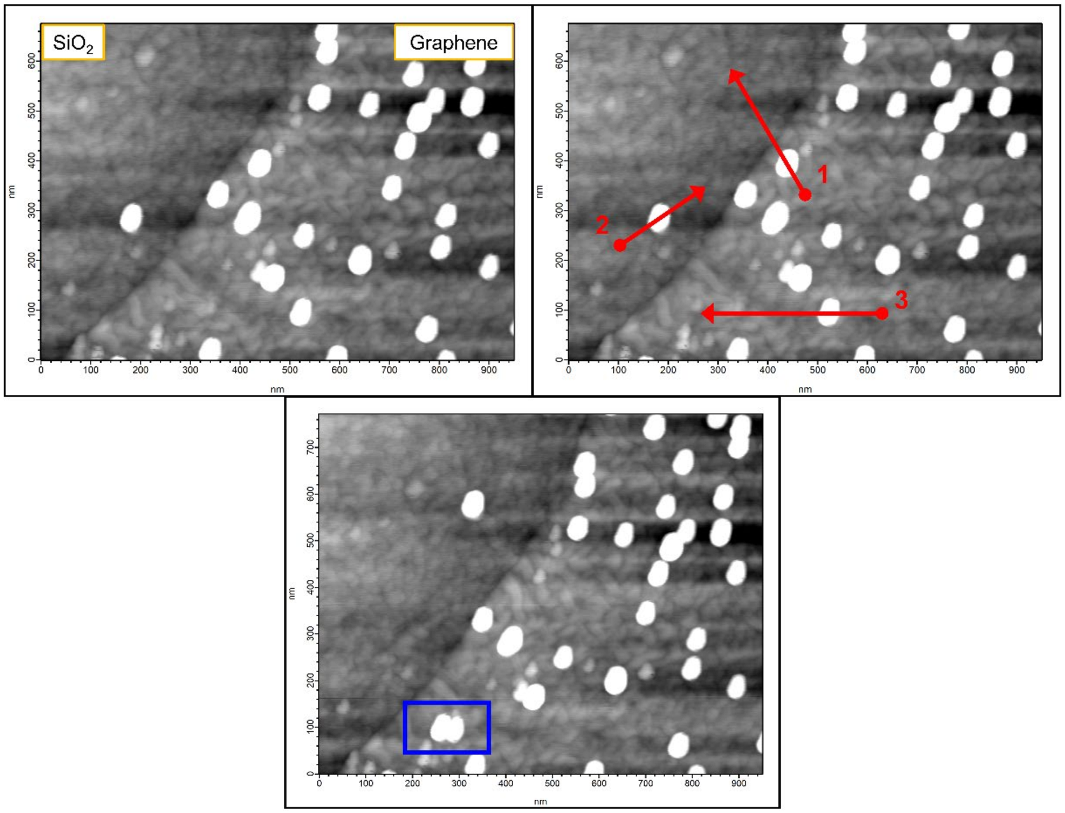

Figure S5. Top left: initial image of nanoparticles deposited near a graphene-SiO_2 step edge. Top right: the same image overlayed with the defined contact mode traces showing the programmed particle manipulation paths along the surface. Bottom: image of the outcome of the traced paths.

S-VI. Computational details

The calculations are carried out within the density functional theory (DFT) framework using the Vienna *ab initio* simulation package (VASP)[S3] which employs periodic boundary conditions and plane-wave basis sets. The projector augmented wave (PAW)[S4] method is used to describe the electron-ion interactions, and the Perdew-Burke-Ernzerhof (PBE)[S5] functional is applied to account for the electronic exchange-correlation interactions. A uniform 1×1×1 Monkhorst-Pack *k*-point sampling[S6] and a plane-wave energy cutoff of 400 eV are utilized. The van der Waals interactions are described using the Grimme DFT-D3 method with the Becke-Johnson damping.[S7] The geometry optimization is considered converged when ion forces become less than 10^{-3} eV·Å^{-1}. The SiO_2 surface contains silanol groups (Si-OH) forming a zigzag hydrogen bonded network. A 20 Å vacuum layer is added to the surface normal in all systems to avoid spurious interactions between the periodic images.

S-VII. References

(S1) Senden, T. J.; Ducker, W. A. Surface Roughness of Plasma-Treated Mica. *Langmuir* **1992**, *8*, 733-735.

(S2) Ostendorf, F.; Schmitz, C.; Hirth, S.; Kühnle, A.; Kolodziej, J. J.; Reichling, M. How Flat is an Air-Cleaved Mica Surface? *Nanotechnology* **2008**, *19*, 305705-305705.

(S3) Kresse, G.; Furthmüller, J. Efficient Iterative Schemes for *Ab Initio* Total-Energy Calculations Using a Plane-Wave Basis Set. *Phys. Rev. B* **1996**, *54*, 11169-11186.

(S4) Blöchl, P. E. Projector Augmented-Wave Method. *Phys. Rev. B* **1994**, *50*, 17953-17979.

(S5) Perdew, J. P.; Burke, K.; Ernzerhof, M. Generalized Gradient Approximation Made Simple. *Phys. Rev. Lett.* **1996**, *77*, 3865-3868.

(S6) Monkhorst, H. J.; Pack, J. D. Special Points for Brillouin-Zone Integrations. *Phys. Rev. B* **1976**, *13*, 5188-5192.

(S7) Grimme, S.; Antony, J.; Ehrlich, S.; Krieg, H. A Consistent and Accurate *Ab Initio* Parametrization of Density Functional Dispersion Correction (DFT-D) for the 94 Elements H-Pu. *J. Chem. Phys.* **2010**, *132*.
